# Enhancing Cellular Interactions Through Bioactivation and Local Nanomechanical Reinforcement in Nanodiamond-Loaded 3D-Printed Gellan Gum Scaffolds

**DOI:** 10.3390/ma18174131

**Published:** 2025-09-03

**Authors:** Carmen-Valentina Nicolae, Masoumeh Jahani Kadousaraei, Elena Olăreț, Andrada Serafim, Mehmet Serhat Aydin, Ioana-Teodora Bogdan, Adriana Elena Bratu, Raluca-Elena Ginghină, Alexandra Dobranici, Sorina Dinescu, Kamal Mustafa, Izabela-Cristina Stancu

**Affiliations:** 1 Advanced Polymer Materials Group, Faculty of Chemical Engineering and Biotechnologies, National University of Science and Technology Politehnica Bucharest, 011061 Bucharest, Romania; carmen.nicolae@upb.ro (C.-V.N.); elena.olaret@upb.ro (E.O.); andrada.serafim0810@upb.ro (A.S.); adriana.elena1930@gmail.com (A.E.B.); 2 Center of Translational Oral Research, Department of Clinical Dentistry, University of Bergen, 5009 Bergen, Norway; masoumeh.kadousaraei@uib.no (M.J.K.); mehmet.aydin@uib.no (M.S.A.); kamal.mustafa@uib.no (K.M.); 3 Faculty of Medical Engineering, National University of Science and Technology Politehnica Bucharest, 011061 Bucharest, Romania; ioana.bogdan@stud.fim.upb.ro; 4 Research and Innovation Center for CBRN Defense and Ecology, 041327 Bucharest, Romania; ginghinaraluca@gmail.com; 5 Department of Biochemistry and Molecular Biology, Faculty of Biology, University of Bucharest, 050095 Bucharest, Romania; dobranici.alexandra-elena@s.bio.unibuc.ro (A.D.); sorina.dinescu@bio.unibuc.ro (S.D.); 6 The Research Institute of the University of Bucharest, 050663 Bucharest, Romania

**Keywords:** 3D printing, gellan gum, nanodiamond nanoparticles, bioactivation, icariin

## Abstract

The integration of nanomaterials within hydrogel scaffolds offers significant promise in bone tissue engineering by improving mechanical performance and modulating cellular responses through mechanotransductive and biochemical signaling. Previous studies have demonstrated that nanodiamonds (NDs) incorporated in electrospun microfibrillar meshes enhance cellular adhesion, spreading, and cytoskeletal organization through localized mechanical reinforcement. However, the effects of ND loading into soft, bioinert three-dimensional hydrogel matrices remain underexplored. Here, we developed nanostructured 3D printing inks composed of gellan gum (GG) supplemented with a low content of ND nanoadditive (0–3% *w*/*v*). ND integration improved the shear-thinning properties of the formulation, enabling consistent filament formation and reliable extrusion-based 3D printing. Structural and mechanical assessments confirmed enhanced scaffold morphology, reduced deformation, and improved morphostructural integrity under compression and increased local stiffness at 2% ND loading (GG_ND2%). Biological assessments revealed that increasing ND content enhanced murine preosteoblast viability, proliferation, and attachment, particularly in GG_ND2%. Furthermore, bioactivation of the GG_ND2% formulation with icariin (ICA), a bioflavonoid known for its osteogenic and angiogenic activity, amplified the beneficial cellular responses of MG-63 cells to ND loading, promoting enhanced surface mineralization and improved cell–matrix interactions. Collectively, these findings highlight the potential of ND-reinforced GG scaffolds bioactivated with ICA, integrating structural reinforcement and biological functionalities that may support osteogenic responses.

## 1. Introduction

Composite materials have revolutionized tissue engineering by enabling the design of scaffolds that integrate mechanical support and bioactive functionalities. In particular, the integration of nanomaterials into hydrogel matrices creates structures with customizable properties tailored to mimic the nanostructured extracellular matrix of hard tissues, promoting cell adhesion, proliferation, and differentiation [1]. By incorporating nanostructures into carbohydrate matrices such as gellan gum, alginate, or cellulose, the composite systems achieve enhanced rheological properties, mechanical stability, and bioactivity, making them suitable candidates for advanced biomedical applications [2,3].

Nanodiamonds (NDs) have attracted significant attention due to their unique combination of exceptional mechanical properties, high surface area, biocompatibility, and rich surface chemistry [4]. The intrinsic stiffness of the ND core, along with the carboxyl and hydroxyl groups on their surface that enable stable hydrogen bonding with polymeric matrices, make them suitable for reinforcing hydrogel systems [5]. Furthermore, NDs have been shown to enhance scaffold stiffness, improve cell adhesion, and influence cellular behavior by modulating the mechanical and biochemical environment [6]. Recent studies emphasize that substrate stiffness can modulate osteogenic differentiation by regulating mechanotransduction signaling pathways and cytoskeletal organization, thereby influencing osteoblast function and extracellular matrix (ECM) deposition, a process also observed in some nanostructured systems [7,8]. Mechanical stimulation has been shown to induce translocation of certain regulatory proteins from the cell cytoplasm into their nucleus, where they interact with transcription factors and modulate gene expression and signaling pathways associated with osteogenic differentiation [9,10]. Prior research suggests that ND-induced local stiffening may enhance cell–scaffold interactions, potentially contributing to this mechanotransductive response. Enhanced preferential cellular adhesion contacts were observed in our previous research [11,12,13], suggesting improved cellular interactions (human adipose stem cells (hASCs), fibroblasts, osteoblasts, neural precursors) with locally exposed ND nanoparticles embedded in electrospun gelatin substrates. By providing localized reinforcement, facilitating cell adhesion, and activating mechanotransduction pathways, NDs could modulate cellular forces and biochemical signaling to create microenvironments conducive to osteogenic differentiation. Although low concentrations of NDs have been shown to promote cellular adhesion and local stiffness in electrospun microfibrillar gelatin meshes, the effects of ND incorporation within soft three-dimensional matrices lacking cell-adhesive moieties remain underexplored. To bridge this gap, we have designed and evaluated novel nanostructured hydrogel systems by integrating low concentrations of NDs (0–3%) into a bioinert gellan gum matrix.

Gellan gum (GG), a water-soluble polysaccharide derived from renewable biomass, particularly strains of Sphingomonas elodea bacteria, is recognized for its gel-forming ability, biocompatibility, and printability [14]. However, it lacks cell-recognition motifs, and therefore, its ability to actively guide cellular responses remains limited, with various biofunctionalization strategies often being employed to improve the biological performance of GG-based scaffolds [15]. In this work, we investigated the potential enhancement of the biological performance of GG_ND composite systems through bioactivation with icariin (ICA), a bioflavonoid known for its osteogenic, angiogenic, and anti-inflammatory properties [16,17]. We reasoned that decorating NDs with ICA allows for complementary enhancement of scaffold properties, combining the mechanical benefits of the nanoadditive reinforcement with the biological activity of ICA [18]. This approach aims to fabricate scaffolds capable of addressing the complex requirements of bone tissue engineering, particularly the need for materials that support both structural integrity and biological functionality.

Prior studies have explored NDs as drug carriers, imaging agents, nanoscale structural reinforcements, or biosensors, typically embedded in matrices such as gelatin, poly(lactic-co-glycolic acid) (PLGA), or polycaprolactone (PCL) [4,5,11,19,20]. Separately, ICA has been loaded into scaffolds based on calcium phosphates, natural polymer blends, or synthetic polymers like PLGA and PCL, owing to its osteogenic and angiogenic bioactivity [21,22,23,24]. ICA’s favorable properties for osteochondral and bone tissue regeneration have been reviewed by Oprita et al. [25], reporting that it promotes bone formation, counteracts bone loss, supports mineralization, and helps prevent estrogen deficiency-related fractures by activating estrogen receptors.

Given their complementary advantages, combinations of ICA and NDs have been recently explored. ICA-functionalized NDs have been studied for enhancing osteogenesis and reducing inflammation [18,26]. However, these systems were limited to nanoparticle dispersions or 2D cell culture platforms and lacked integration into printable composite formulations. To date, no published work has reported the integration of ICA-functionalized NDs into a hydrogel system nor their use in extrusion-based 3D-printed scaffolds. Our study aims to bridge this gap by proposing the first printable, bioactive composite formulations combining GG, NDs, and ICA for structural reinforcement and enhanced cellular interactions for bone regeneration.

Therefore, this study focuses on the development and evaluation of nanostructured GG_ND composite scaffolds, with the objective of enhancing printability, mechanical performance, and biological functionality. By investigating the effects of incorporating low concentrations of NDs on the rheological properties, on filament stability, and on reinforcement of the GG matrix, structural advantages of ND loading in 3D-printed scaffolds are explored. The cellular behavior of preosteoblasts and osteoblast-like cells in response to ND-loaded hydrogels, both before and after ICA biofunctionalization, is investigated. By integrating structural reinforcement and bioactivation, this study provides insights into the design of multifunctional nanocomposite scaffolds for bone tissue regeneration.

## 2. Materials and Methods

### 2.1. Ink Preparation

**Figure 1:**
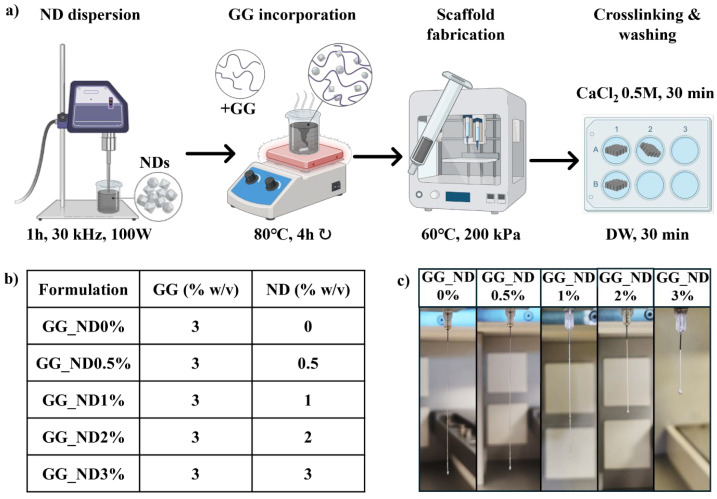
Preparation and filament formation of GG_ND inks: (**a**) schematic overview of the preparation of nanostructured ink formulations; (**b**) composition of the five inks with varying ND concentrations from 0 to 3% *w*/*v*; (**c**) representative photographs from the filament drop test.

Five injectable nanostructured formulations were developed based on a 3% *w*/*v* GG matrix (Phytagel^TM^, P8169, BioReagent, powder, Sigma-Aldrich, Burlington, MA, USA) loaded with low concentrations of NDs (diamond nanopowder, 636444, <10 nm particle size, Sigma-Aldrich), ranging from 0 to 3% *w*/*v*. For each formulation, the ND powder was dispersed in sterilized distilled water (DW) using an ultrasonic processor (UP100H, Hielscher Ultrasonics GmbH, Teltow, Germany; 100 W, 30 kHz, cycle 1, 100% amplitude) for 1 h, ensuring homogeneous nanoparticle dispersion (Figure 1a). To maintain a low temperature and prevent evaporation, the process was carried out in an ice bath. Subsequently, the ND dispersion was heated to 80 °C, and GG was gradually added under continuous magnetic stirring for 4 h to ensure complete solubilization of the polysaccharide while avoiding evaporation. The formulations (detailed in Figure 1b) were stored at 4 °C overnight for subsequent testing and scaffold fabrication. The control was prepared using the same procedure, without the addition of ND. All formulations were prepared following consistent protocols, with no observable batch-to-batch variations.

### 2.2. Rheological Characterization

The rheological properties of the developed formulations were evaluated using a Kinexus Pro rheometer (Malvern Instruments, Worcestershire, UK) equipped with a parallel plate configuration and a Peltier cell to ensure precise temperature control. The shear-thinning properties of the materials were explored by measuring their viscosity across a broad range of shear rates (0.01–1000 s^−1^). To investigate their thixotropic recovery properties, a three-interval thixotropy test (3ITT) was performed, simulating the different stages encountered within the material before, during, and after the extrusion-based fabrication process [27]. In the first interval, the inks were subjected to low shear rates (0.1 s^−1^ for 120 s), representing the pre-printing stage, when materials are stored in the cartridge. During the second interval, high shear rates (100 s^−1^ for 60 s) were applied to simulate the forces induced within the material during extrusion. The third interval replicates post-extrusion conditions, exposing the materials to low shear rates to evaluate viscosity recovery. All measurements were conducted in triplicate at the printing temperature (60 °C), using a solvent trap to prevent dehydration.

The acquired rheological data were fitted to two mathematical models commonly used to describe non-Newtonian fluids: the power law model [27] (Equation (1)) and Carreau [28] (Equation (2)). The models were selected for their accuracy in describing the non-linear relationship between applied shear rate ( $\dot{\gamma }$ ) and viscosity ( μ ), characteristic of shear-thinning materials.

(1) $$\mathrm{Power}\;\mathrm{law}\;\;\;\;\;\;\;\;\;\;\;\;\;\;\;\;\;\;\;\;\mu_{\infty }=m\cdot {\left(\frac{\dot{\gamma }}{{\dot{\gamma }}_{ref}}\right)}^{n-1}$$

(2) $$\mathrm{Carreau}\;\;\;\;\;\;\;\;\;\;\;\;\;\mu {=\mu }_{\infty }+(\mu_{0}-\mu_{\infty }){\left(1+\lambda {\dot{\;\gamma }}^{2}\right)}^{\frac{n-1}{2}}$$

The power law model employs two key parameters, m (consistency index) and n (flow behavior index), defining viscosity relative to a reference shear rate, ${\dot{\gamma }}_{ref}$ , which is typically set to 1 s^−1^. When n < 1, the model describes shear-thinning characteristics, whereas n = 1 describes Newtonian fluid behavior. The Carreau model accounts for the materials’ viscosities at both zero and infinite shear rates, represented as $\mu_{0}$ and $\mu_{\infty }$ , taking into account the fluid relaxation time ( λ ) to describe the transition between the two viscosities under varying shear rates, along with the n exponent to indicate the degree of shear thinning. The model parameters were determined by fitting the experimental rheological data through least-squares regression using a custom MATLAB (version R2023a) script.

Computational fluid dynamics (CFD) simulations were conducted to analyze the flow behavior of the formulations during extrusion-based 3D printing, assessing parameters such as shear rate, wall shear stress, pressure distribution, and dynamic viscosity within the cartridge and needle. The experimental setup geometry was recreated in SolidWorks 2021 (Dassault Systèmes, Vélizy-Villacoublay, France), with respect to the dimensions and configurations of the cartridge and the needle. Material parameters such as density and viscosity were assigned based on experimental measurements for each composition, considering the viscosity dependence on temperature and shear rate. Mathematical fitting to the power law model provided the consistency coefficients and power law index of the developed formulations, derived from rheological data. Simulations were designed to replicate realistic printing conditions and process parameters, including temperature (60 °C), applied pressure (200 kPa), and extrusion velocity (10 mm/s). Gravity (9.81 m/s^2^) was accounted for, while surface roughness and wall slip effects were assumed negligible, under adiabatic wall boundary conditions.

### 2.3. Scaffold Fabrication

The injectability of the formulations was preliminarily assessed using a 1 mL syringe equipped with a 0.2 mm inner diameter metallic needle to evaluate the ease of extrusion through the printing nozzle. The suitable materials were further loaded into 10 mL plastic cartridges and subsequently mounted onto the extrusion-based printhead (Direct Dispensing) of the 3D printing equipment (3D Discovery, RegenHu, Villaz-Saint-Pierre, Switzerland). Prior to printing, the formulations were brought to 60 °C using a heating mantle for 1 h to ensure homogeneous temperature distribution within the cartridge, and filament formation was assessed. Scaffolds were printed based on a 10 × 10 mm grid design, with 2.5 mm filament spacing and a 90° orientation offset between consecutive layers. Printing was performed at a speed of 10 mm/s, with an extrusion pressure of 200–220 kPa. The formulations were extruded through a 0.2 mm diameter metallic needle onto glass slides cleaned with a 70% (*v*/*v*) ethanol solution.

Post-printing, scaffolds were stabilized through ionic crosslinking of GG, using divalent calcium ions. Each printed structure (10 × 10 × 8 mm) was immersed in 3 mL 0.5 M CaCl_2_ solution for 30 min to allow cation diffusion within the scaffold matrix. After crosslinking, the structures were thoroughly washed with DW to remove soluble fractions and excess calcium ions. The stabilized scaffolds were either used as-printed or dried by sequential immersion in ethanol solutions of increasing concentrations (20% to 100%) followed by oven drying overnight at 37 °C. The dry scaffolds were stored in airtight tubes for subsequent characterization.

### 2.4. Hydrogel Characterization

The mechanical properties of the hydrogels were evaluated to determine the reinforcing effect of the ND particles on the GG matrix. In this regard, coin-shaped samples (20 mm in diameter and 1 mm in height) were crosslinked as described above (Section 2.3). Mechanical testing was performed with the Kinexus Pro rheometer, using a plate-plate configuration with roughened surfaces to minimize slippage. The set gap ranged from 0.85 to 0.95 mm, and a solvent trap was used to prevent dehydration of the hydrogel samples during measurements performed at room temperature (25 °C). Oscillatory dynamic tests were conducted at a constant frequency of 1 Hz to determine the linear viscoelastic region (LVR), with stress varying between 0.01 and 1000 Pa. From the LVR, a shear stress value of 1 Pa was selected and maintained during frequency sweep tests, where the frequency was varied from 10 Hz to 0.1 Hz.

The influence of the ND loading on the morphology of the scaffolds was evaluated through scanning electron microscopy (SEM). Dried printed samples were sputter-coated with a thin layer of gold (DSR1, Vac Techniche, East Sussex, UK) and analyzed using high-resolution TESCAN CLARA SEM (Brno, Czech Republic) at an accelerating voltage of 1 kV. Representative pore area fraction, expressed as the ratio of total pore area to total image area, along with average pore area, was quantified from SEM top-view micrographs using ImageJ software (version 1.54i).

To examine the morpho-structural deformation induced by uniaxial compression on the scaffold architecture at varying NDs concentrations, printed scaffolds were hydrated overnight and subsequently compressed to 40% deformation using a high-resolution computerized micro-tomograph (µCT, Skyscan 1272, Bruker, Kontich, Belgium) equipped with a Material Testing Stage (MTS440, Bruker, Kontich, Belgium). The compression force applied was 440 N, while the projection set was recorded at a resolution of 2452 × 1640 pixels using a 0.5 mm Al filter. Scanning was conducted at 100 kV intensity and 55 µA, achieving a pixel size of 0.8 µm for detailed imaging. The samples were rotated 360°, with projections acquired at 0.2° intervals, at an exposure time of 250 ms per projection. The collected data were reconstructed using CTRecon software (version 1.7.1.6), and the resulting cross-sectional slices were employed for 3D visualization with CTVox software (version 3.3.0 r1403). The compressive modulus of the scaffolds was determined through unconfined uniaxial compression using a Brookfield CT3 Texture Analyzer (Brookfield Engineering Laboratories Inc., Middleboro, MA, USA) equipped with a 4500 g load cell and a TA25/1000 cylindrical probe (Brookfield Engineering Laboratories Inc.). Hydrated printed scaffolds (approx. 7 × 7 × 5 mm, measured with a digital caliper) were compressed at a rate of 0.5 mm/s. The compressive modulus was calculated at 2% strain. Data acquisition and analysis were performed using the dedicated TexturePro CT Build 31 software (version 1.8).

The scaffolds’ local nanomechanical properties were investigated through nanoindentation using a G200 Nanoindenter (KLA instruments, Milpitas, CA, USA) equipped with a Dynamic Contact Module II (DCM II) indentation head (KLA instruments). Printed hydrogel samples were hydrated overnight at 37 °C prior to testing. Oscillatory indentation was performed to a depth of 10 µm at a frequency of 10 Hz using a cylindrical flat punch indenter with a diameter of 511.36 µm. The storage modulus (G′) and loss modulus (G″) were determined using the G-series DCM CSM Flat Punch Complex Modulus Method, as implemented in the equipment’s Nanosuite software (version 6.52).

In order to assess GG_ND substrates’ in vitro biocompatibility and ability to support cell adhesion, MC3T3-E1 (ATCC, CRL-2593, Manassas, VA, USA) murine preosteoblast cell line was used. MC3T3-E1 cells were grown and expanded in culture according to manufacturer’s instructions in Dulbecco’s Modified Eagle Medium (DMEM, Sigma/Merck, Steinheim, Germany) supplemented with 10% fetal bovine serum (FBS) (Thermo Fisher Scientific, Grand Island, NY, USA), 1% L-glutamine, sodium pyruvate, and antibiotics (P/S, Penicilin/Streptomycin, Sigma-Aldrich, St. Louis, MO, USA). Cells were seeded onto GG_ND0%, GG_ND0.5%, GG_ND1%, and GG_ND2% compositions in 48-well culture plates at a density of 1.5 × 10^4^ cells/cm^2^ and maintained in standard culture conditions (37 °C, 5% CO_2_, humidified atmosphere) for up to 7 days. Prior to cell seeding, all scaffolds were UV-sterilized for 6 h per side. Biocompatibility assays were performed after 1 and 7 days of in vitro culture.

The metabolic activity of living preosteoblasts in contact with the GG_ND substrates was quantified using the MTT assay. Tissue culture plastic (TCP) was represented by cells seeded onto 48-well plate and served as a positive control. In this regard, a working solution consisting of methylthiazolyldiphenyl tetrazolium bromide (MTT, Sigma/Merck, Steinheim, Germany) was prepared in serum-free culture medium at a final concentration of 1 mg/mL, according to manufacturer’s instructions. Culture medium containing FBS was removed from the samples, and MC3T3-E1_GG_ND systems were washed with phosphate saline buffer (PBS) and incubated with MTT solution for 4 h at 37 °C. After formazan crystals were formed, they were solubilized with isopropanol, and the resulting violet solution was measured at 550 nm using a FlexStation3 spectrophotometer (Molecular Devices, Foster City, CA, USA).

Lactic dehydrogenase (LDH) test was utilized to quantify any cytotoxic effects of the GG_ND scaffolds using the in vitro toxicology assay kit and lactic dehydrogenase-based TOX7 kit (Sigma-Aldrich). For positive control, cells seeded onto 48-well plate were treated with 2% Triton X-100 solution (Sigma/Merck, Steinheim, Germany). The assay was performed according to manufacturer’s instructions, and the absorbance of the resulting solutions was determined at 490 nm using FlexStation3 spectrophotometer (Molecular Devices, Foster City, CA, USA).

Further, in order to simultaneously observe the live and dead cells in contact with the tested compositions, we used a Live/Dead kit (Invitrogen, Life Technologies, Foster City, CA, USA) that utilizes calcein AM to stain living cells and ethidium bromide (EtBr) to stain dead cells’ nuclei. The working solution was prepared according to manufacturer’s instructions, added to each sample, and incubated at room temperature in the dark for 30 min. After incubation time, sample investigation was performed with a laser-scanning confocal microscope (Nikon A1/A1R Confocal Laser Microscope System), and the acquired images were analysed with the corresponding software.

To investigate cellular adhesion onto the GG_ND substrates, cytoskeleton and focal adhesion staining were performed. After 48 h post-seeding, MC3T3-E1_GG_ND systems were fixed using a 4% paraformaldehyde (PFA) solution (Sigma-Aldrich Co., Steinheim, Germany). Upon fixation, samples were washed with PBS and permeabilized for 20 min with 2% bovine serum albumin and 0.1% Triton X-100 solution. Samples were then incubated overnight at 4 °C with a solution of phalloidin-FITC (Sigma/Merck, Steinheim, Germany). After that, cells’ nuclei were stained with Hoechst 33342 (Thermo Fisher Scientific, Foster City, CA, USA) for 10 min at room temperature in the dark. Samples were visualized with the same laser scanning confocal microscope used for Live/Dead staining, and image analysis was performed with the same software. Quantitative image analysis was performed using ImageJ software (version 1.54i). Cell area measurements were conducted on phalloidin-stained images, while fluorescence intensity was quantified to assess actin filament density.

### 2.5. Bioactivation

Based on prior assessments, the GG_ND2% formulation was selected for further bioactivation using ICA. A total of 10 mg of ICA powder (Sigma-Aldrich), corresponding to a final concentration of 0.1% *w*/*v* in the ink, was first dissolved in 100 µL dimethyl sulfoxide (DMSO, Sigma). After ICA solubilization, the resulting ICA-DMSO solution was added to sterile DW under magnetic stirring, resulting in a final 1% *v*/*v* DMSO concentration. Subsequently, ND powder was added and further dispersed through sonication for 1 h. GG was gradually incorporated as previously described (Section 2.1), forming the bioactivated formulation (GG_ND2%_ICA). To provide comparative controls, two additional formulations were similarly prepared—a negative control containing 3% GG and 2% NDs (GG_ND2%) and a positive control consisting of 3% GG supplemented with 0.1% ICA (GG_ICA). Hydrogel casts for all formulations were molded into 5 mm diameter discs, crosslinked with CaCl_2_ for 30 min, washed, and subsequently used to evaluate the potential chemical interactions between the components, as well as the potential enhancements in biological performance induced by ICA.

The compositional characterization of the raw materials (GG, NDs, ICA) and selected formulations (GG_ND2%, GG_ICA, and GG_ND2%_ICA) was performed by attenuated total reflectance Fourier-transform infrared spectroscopy (ATR-FTIR) in order to assess the possible physical interactions between ICA and ND particles within the composite formulations. A Jasco 4200 spectrometer (JASCO, Tokyo, Japan) equipped with a Golden Gate ATR module (Specac Ltd., Orpington, UK) was used to acquire spectra in the range of 4000–600 cm^−1^ at a resolution of 4 cm^−1^.

To evaluate the biological response to the ICA-laden hydrogels, osteoblast-like cells (MG-63, CRL-1427^TM^, ATCC, Glasgow, UK) were cultured in complete medium (CM), consisting of DMEM (4.5 g/l glucose, Life Technologies, Carlsbad, CA, USA) supplemented with 1% antibiotic (P/S, SV30010, cytiva HyClone, 10 000 U/mL; Thermo Fisher Scientific, Logan, UT, USA) and 10% FBS (A5256701, Thermo Fisher Scientific, Waltham, MA, USA), under standard conditions. Cells were expanded until appropriate confluency and subsequently trypsinized, counted, and seeded onto 5 mm diameter hydrogel scaffolds at a density of 20 × 10^4^ cells per scaffold. They were cultured for 7 days in CM supplemented with 100 μg ml^−1^ antifungal (Normocin^TM^ Antimicrobial reagent, InvivoGen, San Diego, CA, USA), with medium changes every 2–3 days.

MG-63 viability was assessed on days 1 and 7 of culture. Scaffolds were washed with Dulbecco’s Phosphate-Buffered Saline (DPBS, Gibco, Thermo Fisher Scientific) and stained using a Live/Dead staining kit (L3224, Invitrogen^TM^, Thermo Fisher Scientific), according to the manufacturer’s instructions. Samples were visualized under fluorescence microscopy using Andor Dragonfly 5050 high-speed confocal microscope (Oxford Instruments, Belfast, UK) to evaluate cell viability and distribution on the scaffolds. Images were processed using Imaris software (version 9.9.1, Oxford Instruments, Belfast, UK).

Morphostructural changes induced on the scaffold surface post-cell culture were observed after 1 and 7 days of MG-63 culture. Cells were fixed with 4% *w*/*v* PFA, and the scaffolds were dried for 20 min at room temperature and dehydrated through sequential immersion in ethanol solutions, followed by overnight drying at room temperature. Further, scaffolds were sputter-coated with a thin layer of gold and imaged using high-resolution SEM (Leo Supra VP 55, Zeiss, Oberkochen, Baden-Württemberg, Germany) at an accelerating voltage of 10–15 kV and 5–7 mm working distance.

### 2.6. Statistical Analysis

Statistical analyses were performed using GraphPad Prism (v8.02 or 8.4.3, GraphPad Software Inc., San Diego, CA, USA). Data normality was assessed using the Shapiro–Wilk test. For datasets that met the assumptions of normality, one-way or two-way ANOVA followed by Tukey’s or Bonferroni’s post hoc tests was applied. Otherwise, Welch’s ANOVA was performed, with Brown–Forsythe’s test included for homoscedasticity and Tamhane T2 post hoc test included for multiple comparisons. All experiments were conducted with a minimum of three replicates, and results are presented as mean ± standard deviation. Statistical significance was defined as *p* < 0.05. Non-significant comparisons were left unmarked.

## 3. Results and Discussions

### 3.1. Ink Preparation and Rheological Behavior

An overview of the ink development process is illustrated in Figure 1a, showing the five GG-based nanostructured formulations prepared with ND loadings ranging from 0 to 3% *w*/*v* (Figure 1b). Ultrasonication effectively dispersed the ND powder before the stepwise addition of GG powder at 80 °C, while the prolonged preparation time (4 h under magnetic stirring, covered to prevent evaporation) ensured ink homogeneity. GG is often used as a versatile material for extrusion-based 3D printing and bioprinting applications due to its excellent printability, ease of processing, and mild ionic gelation conditions [29]. In this study, GG was selected since it is widely used in tissue engineering, while it is inherently bioinert, with limited cellular interactions, providing a matrix for exploring the potential of ND to stimulate cell interactions. By varying the ND concentration within this soft, inert matrix, these formulations were designed to determine whether ND loading could induce changes in the local microenvironment and subsequently modulate cellular responses in hydrated 3D systems.

The development of the inks produced smooth precursor solutions for ND concentrations up to 2%, all of which could be extruded through the 0.2 mm metallic needle and crosslinked by CaCl_2_ immersion to form robust filaments. The injectability and extrusion behavior of the formulations were first evaluated manually, using a 1 mL syringe equipped with a printing needle. All formulations formed continuous filaments upon deposition on a glass slide, indicating baseline suitability for printing. Filament formation under 3D printing conditions was further evaluated by employing the filament drop test [30,31], where filaments were extruded freely into the air to assess their flow characteristics and filament structural integrity (Figure 1c). An optimal formulation should produce a continuous, uniform filament that ensures stable and reproducible extrusion. Conversely, formulations with insufficient viscosity tend to form droplets, unable to support subsequent scaffold layers, while overly viscous inks lead to discontinuous filament geometry due to impaired material flow.

The incorporation of NDs notably influenced continuous filament formation at 1 and 2%. GG_ND0% and GG_ND0.5% formed irregular filaments, with structural inconsistencies resembling filaments previously described as over-gelled by Ouyang et al. [31]. As ND concentration increased, filament uniformity was visibly improved. GG_ND1% and GG_ND2% produced well-defined, continuous filaments with consistent diameters, demonstrating an adequate balance between viscosity and filament formation ability. In contrast, GG_ND3% frequently caused needle clogging, generating irregular, discontinuous filaments upon extrusion, likely due to nanoparticle aggregation within the needle lumen. These aggregation phenomena often disrupted extrusion, making the GG_ND3% formulation unreliable for extrusion under similar conditions. In fibrous electrospun poly(vinyl alcohol) loaded with ND concentrations ranging from 0 to 5% *w*/*v*, Wang et al. [32] observed that their dispersion in water, applied in a similar manner as in our study, was most effective for the 2% ND loading. This led to an increased reinforcing effect for the 2% ND loading compared to other ND concentrations. The 2% ND formulation yielded stronger electrospun mats with increased Young′s modulus, tensile strength, and elongation at break without impeding the fabrication process. However, higher concentrations of NDs reduced the tensile modulus and tensile strength of the nanofibrous materials. Taken together, these findings, our own processing experience, and the fabrication process limitations led us to select four formulations containing 0–2% *w*/*v* NDs for further characterization.

**Figure 2:**
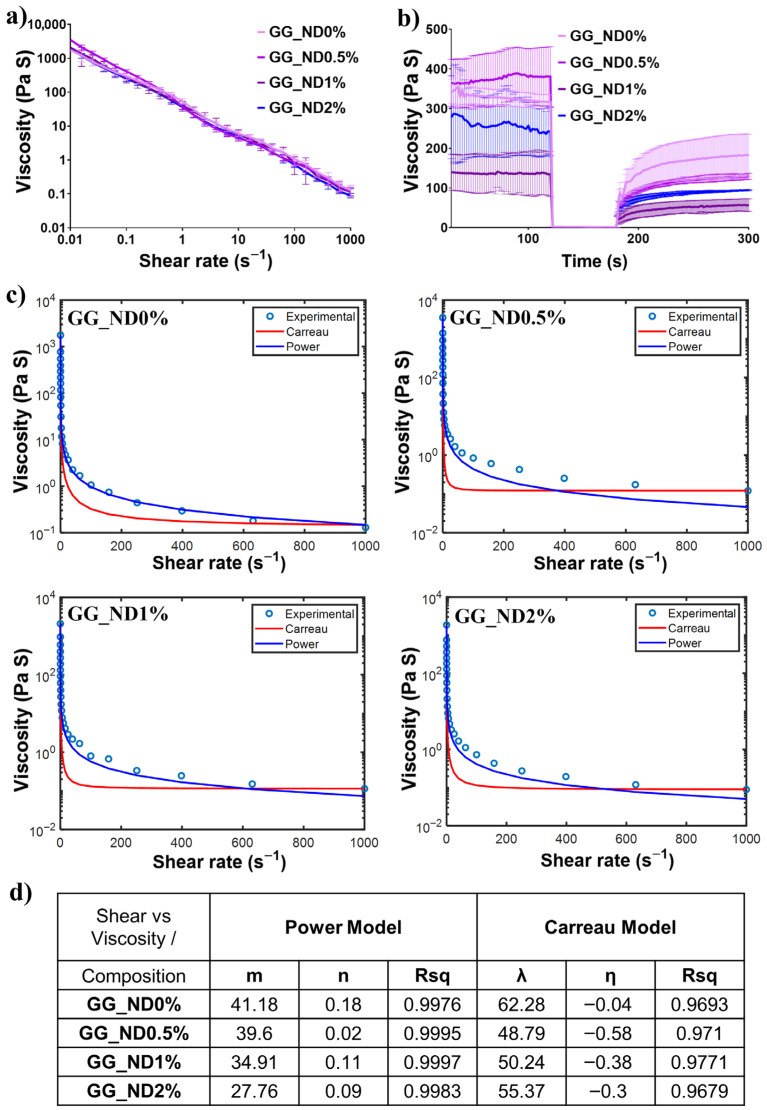
Rheological characterization and mathematical modelling of GG_ND inks: (**a**) viscosity versus shear rate curves; (**b**) three-interval thixotropy (3ITT) test; (**c**) rheological data fitted with the power law and Carreau models for each composition; (**d**) fitting parameters obtained for the two mathematical models.

Subsequently, the rheological properties of the four selected formulations were assessed to evaluate the effect of increasing ND loading on ink viscosity, shear-thinning behavior, and viscosity recovery post-extrusion. All tested formulations exhibited pronounced shear-thinning behavior, as evidenced by the progressive decrease in viscosity observed with increasing shear rate (Figure 2a). GG′s inherent shear thinning properties, along with the colloidal characteristics of NDs, effectively facilitated filament continuity and consistency during extrusion, particularly for inks containing 1 and 2% *w*/*v* NDs. While all formulations exhibited similar viscosity decreasing curves, GG_ND2% consistently showed lower values across the tested shear rate interval. This behavior suggested improved fluidity under stress, which could indicate balanced particle distribution and reduced aggregation on one hand, while their presence between polymer chains could moderately disrupt GG polymer–polymer interactions on the other hand, both of which enhance shear thinning properties. This non-Newtonian, pseudoplastic behavior is essential for extrusion-based fabrication processes, as it facilitates material flow through the nozzle under applied shear stress [27].

Complementarily, after shear thinning supports material flow during extrusion, the inks’ rapid viscosity recovery post-extrusion ensures filament stability, contributing to their ability to support multiple layers and, ultimately, to the shape fidelity of the printed objects. Through three-interval thixotropy tests [33], the stress applied to the material simulates material storage within the cartridge, where high viscosity is maintained (Figure 2b, first interval, 120 s). Further, applied shear stress increases, reflecting the conditions induced within the material during extrusion (second interval, 60 s). All formulations showed a rapid decrease in viscosity, ranging from a 90% reduction for GG_ND0% to an over 99.5% reduction in formulations containing NDs. The increased viscosity reduction in ND-containing formulations could indicate that ND incorporation improves material flow during extrusion, consistent with other nanostructured bioink systems, where nanoparticles were shown to reduce internal friction, enhance shear thinning, and improve extrusion behavior [34]. Post-extrusion, the viscosity recovery rates were maintained between 30 and 35% for all inks, indicating partial structural recovery (third interval, 120 s). While the literature typically reports higher recovery rates, typically over 80% [27] for maintaining shape fidelity, it is important to note that this benchmark is not absolute and remains highly dependent on the specific ink composition and associated physicochemical properties. In our case, despite moderate viscosity recovery rates, the printed filaments and structures remained stable and maintained their designed architecture, with each layer effectively supporting subsequent layers (for up to 60 layers) during fabrication. This could be primarily attributed to the inherent thermal gelation properties of GG. The selected printing temperature (60 °C) was determined based on preliminary studies and data in the literature, demonstrating that GG undergoes a primary to secondary structure transition, forming stable junction zones at elevated temperatures and promoting optimal printability and rapid filament stabilization upon cooling [35]. Post-printing, exposure to room temperature induced GG chain entanglement, going from single-stranded chains to double-helix structures [36]. Hydrogen bond formation with incorporated NDs is facilitated by GG′s high molecular weight and the rich chemical functionalities along its polymeric backbone, as well as on the nanofiller surface, effectively contributing to the printed filament stabilization. Therefore, despite the low viscosity recovery values, the consistent absence of structural collapse during multi-layered printing indicates that print fidelity in this system is not exclusively dependent on thixotropic recovery. Although our recovery values (30–35%) were lower than commonly cited thresholds for printable inks, we were unable to identify previous studies that report similarly low viscosity recovery values while achieving comparable high structural fidelity. This suggests that thixotropic recovery thresholds may not be universally applicable, particularly in systems such as ours that could rely on thermal gelation and hydrogen bonding to stabilize the deposited filaments. While the 3ITT test was performed at the printing temperature (60 °C), structural stabilization occurs rapidly upon extrusion due to the lower ambient temperature (25 °C) in the bioprinter cabinet. Thus, material-specific gelation and reinforcement mechanisms should be considered when interpreting rheological recovery values in the context of ink performance.

The acquired experimental rheological data were fitted to both power law and Carreau models to characterize the shear-thinning behavior and viscosity variations of the developed inks across the investigated shear rate range (0–1000 s^−1^). Figure 2c illustrates the viscosity curves obtained experimentally (blue circles), alongside predictions derived from both the power law (blue line) and Carreau (red line) models. All formulations displayed pronounced shear-thinning characteristics, as evidenced by the rapid viscosity decrease occurring predominantly at shear rates below 50 s^−1^, followed by a gradual reduction in viscosity towards a plateau at higher shear rates (100–1000 s^−1^). This behavior reflects the disruption of intermolecular interactions and physical molecular entanglement within the polymeric network, as well as the possible alignment of the polymer chains in the direction of the flow under high shear, which is consistent with other nanocomposite systems designed for bioprinting [34,37]. Figure 2d shows the parameters obtained for the two mathematical models. The power law model accurately captured the viscosity pattern of all tested formulations, with high correlation coefficients (Rsq > 0.997 for all compositions, Figure 2d). Its superior fit emphasized the strongly pseudoplastic nature of the GG-based inks and suggested that the viscosity dependence on shear rate is best described by a two-parameter relationship. The calculated flow behavior index ( n ) indicated pronounced shear-thinning behavior for all compositions, independent of the ND loading within the tested concentration range, suggesting a limited influence of the nanofiller on the fundamental flow behavior of the composition. In contrast, the Carreau model, although commonly applied to characterize complex fluids, showed lower fitting accuracy (Rsq values ranging from 0.9679 to 0.9771). Specifically, the model underpredicted viscosity at low shear rates and deviated from the experimentally acquired data, as observed in all plots, determining its inability to capture the early-stage structural breakdown dynamics of the GG_ND formulations. This underprediction is primarily due to the Carreau model’s global fitting bias toward high-viscosity regions, which dominate the Rsq calculation over the wide dynamic range of the dataset [38]. Such deviations, particularly at low shear rates, are consistent with known limitations of the model in capturing early-stage structural transitions in polymeric fluids, even when overall Rsq values remain high [39].

These observations are consistent with the previous literature on nanostructured inks, with models like power law providing accuracy for strongly shear-thinning inks in practical extrusion-based scenarios [27,40,41]. Therefore, the accurate power law fitting of the experimental rheological data indicates their potential for 3D printing, providing reliable predictive insights into their flow behavior during scaffold fabrication.

**Figure 3:**
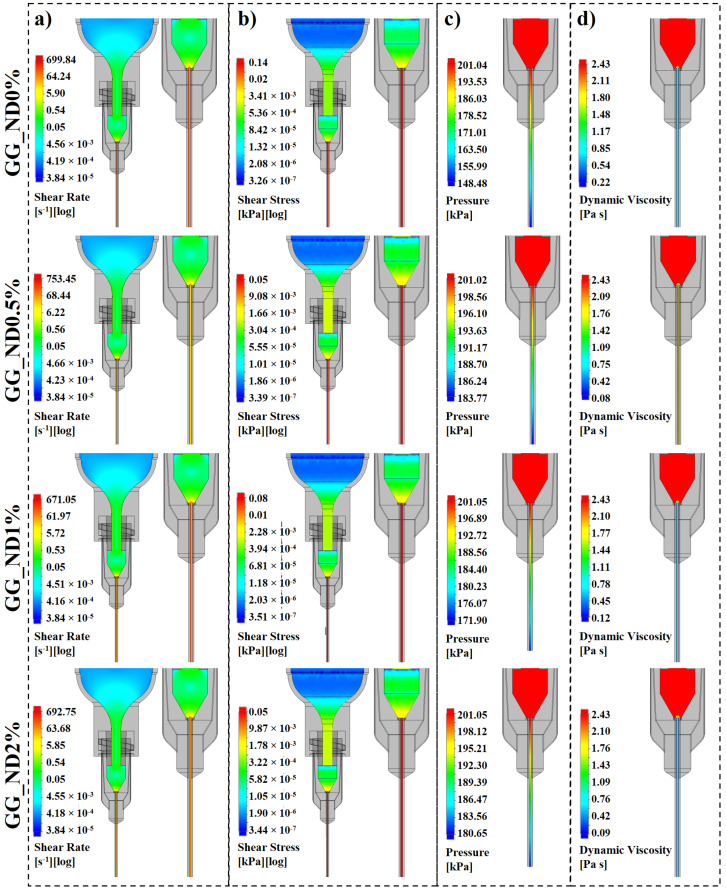
Computer fluid dynamics (CFD) simulations of GG_ND formulations: (**a**) shear rate distribution and (**b**) wall shear stress within the cartridge and the needle, (**c**) pressure gradient, and (**d**) dynamic viscosity profile through the needle.

Experimentally and mathematically obtained parameters, including material densities, viscosity profiles, applied extrusion pressure, and rheological coefficients derived from power law model fitting (Figure 2d), were integrated into CFD simulations (Figure 3). The shear rate (Figure 3a) generated within the cartridge maintained low values for all formulations (3.84 e^−5^ s^−1^), increasing as the geometry narrowed towards the cartridge-needle junction (around 0.5 s^−1^) and particularly towards the needle. The shear rate reached maximum values of 670–750 s^−1^ towards the needle wall, consistent with the literature [42], with gradually lower shear induced towards its lumen (5–200 s^−1^). The mechanical stress that the material experiences during extrusion can cause shear-induced alignment of polymer chains and aid nanoparticle dispersion, reducing viscosity and facilitating the printing process [43], in accordance with our rheological findings. Shear stress (Figure 3b) mirrored the shear rate distribution, concentrating along the inner surface of the needle, where flow resistance is the highest. The stress levels from the cartridge walls (roughly 3.5 × 10^−7^ kPa) to the needle walls (0.14 kPa) remained within a very low range, particularly in formulations containing NDs (0.05–0.08 kPa), indicating a reduced resistance to flow. This slight shear stress attenuation in ND-containing formulations could suggest enhanced nanoparticle–polymer interactions and microstructural rearrangement, which could facilitate chain alignment and lower internal friction under shear without compromising the material integrity [43].

The pressure distribution (Figure 3c) showed a gradient within the needle as the materials transition from the wider chamber of the needle (201 kPa) to the narrow channel within its lumen, dropping to roughly 190 kPa for ND-containing formulations and 148 kPa for GG_ND0% towards the needle tip. The increasing nanofiller content did not disrupt the pressure profile, which remained similar between formulations, confirming that ND incorporation does not compromise printability under consistent conditions. Combined with the mathematical models, rheological and simulated shear data, the CFD simulations mapped out a pronounced shear thinning behavior of the materials, with dynamic viscosity dropping sharply in the high shear regions (Figure 3d) from maximums of 2.43 Pa s within the cartridge to 0.22 Pa s near the needle walls (GG_ND0%). For ND-laden inks, the viscosity reduction is more pronounced, reaching 0.08–0.12 Pa s. While slightly lower than the 99% reduction in viscosity observed in the 3ITT experiments at 100 s^−1^, the values confirm the shear thinning behavior of the compositions, showing a 91% viscosity drop in GG_ND0% and a 95–97% reduction in ND-containing formulations when exposed to shear rates up to 750 s^−1^. Lower viscosity within the needle facilitates material flow, while higher viscosity under low shear suggests the rapid viscosity recovery once extruded, supporting filament shape fidelity post-extrusion. The simulated and experimentally acquired data confirmed that the increasing ND loading did not hinder the reliable extrusion of the materials in consistent printing conditions. On the contrary, the ND content lowered the wall shear stress and enhanced the viscosity reduction under applied shear stress, supporting material flow. In addition, all pressure values were within the practical limits of most extrusion-based 3D printing equipment, making the GG_ND formulations scalable while preventing the need for additional hardware. However, it is important to note that the CFD model used in this study assumed no-slip boundary conditions along the needle and cartridge walls. While this is a common simplification in extrusion-based simulations, particularly when using metallic needles with certain surface roughness, wall slip could influence the flow behavior of shear-thinning materials [40,44,45]. Further, the four selected formulations with 0–2% ND loading were used to fabricate the printed structures, followed by the ionic formation of the GG network using CaCl_2_. After thorough washing, the crosslinked structures were used to assess the reinforcing effect of NDs on the GG matrix, as well as their influence on the biological response.

### 3.2. ND Structural Reinforcing Effect

**Figure 4:**
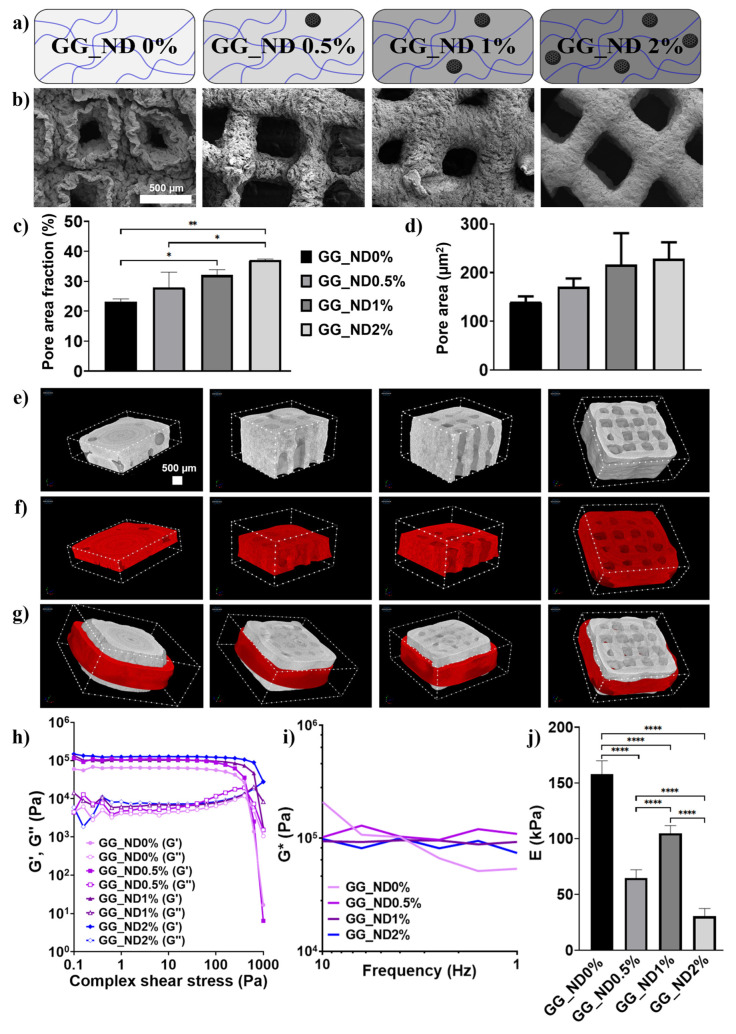
Structural and mechanical reinforcement effects of ND loading in GG scaffolds: (**a**) schematic representation of GG (purple) and NDs (grey) compositions, with increasing concentrations of NDs; (**b**) representative scanning electron microscopy (SEM) micrographs of dry scaffolds, along with their representative (**c**) pore area fraction (%) and (**d**) average pore area (µm^2^) (* *p* < 0.05, ** *p* < 0.01); (**e**–**g**) micro-computed tomography (µCT) reconstructions of hydrated scaffolds (**e**) before compression and (**f**) after 40% uniaxial compression, with (**g**) overlay highlighting deformed regions; (**h**–**i**) oscillatory rheological measurements varying (**h**) the amplitude and (**i**) the frequency of the oscillations; (**j**) compressive modulus (E) of hydrated scaffolds (**** *p* < 0.0001). Scale bars are 500 μm.

The effect of ND loading on the microstructural features of the GG matrix was evaluated through SEM imaging and high-resolution micro-computed tomography (Figure 4). Figure 4a provides a schematic illustration of the polymeric GG matrix with varying concentrations of ND particles (0–2% *w*/*v*). At low concentrations, the nanoparticles are sparsely dispersed, while increased loading leads to more effective nanoparticle–polymer interactions due to improved ND distribution within the matrix. At higher ND concentrations, a certain degree of nanoparticle clustering is anticipated. In this context, the presence of small ND clusters dispersed throughout the macromolecular network is beneficial, as they form stiff nanoscale islands that can act as mechanical anchor points for cell adhesion. This distribution pattern was supported by our previous work, where preferential cell attachment was observed towards ND agglomerates present on electrospun nanofiber surfaces [11,12,13].

The scaffolds’ morphology after dehydration provides indications of the density and stability of the material. In the absence of NDs, the GG matrix had irregular, poorly defined pores and filament structure, as indicated by the SEM images (Figure 4b). The scaffold surface appeared rough and non-uniform, following shrinkage specific to polysaccharide hydrogels, demonstrating instability during the drying process. The addition of 0.5% NDs showed minor improvement in filament definition compared to GG_ND0%, with persistent surface irregularities, filament fusion, and partial collapse. However, increasing the ND content to 1% enhanced filament uniformity, surface smoothness, and pore definition, reducing the previously noticed shrinkage during dehydration. At the highest concentration (2% NDs), the scaffold had an adequate morphological uniformity, well-defined pore architecture, and continuous smooth filament surfaces, indicating the significant structural reinforcement of the NDs. These effects could be attributed to improved nanoparticle–polymer physical interactions, most probably due to H-bonding, and increased solid content, reducing filament collapse and enhancing scaffold robustness during the drying process.

Pore area fraction and average pore size area measurements (Figure 4c,d) reinforce the morphological trends observed in SEM imaging. Increasing the ND content significantly increased the pore area fraction of the scaffolds (Figure 4c), rising from approximately 23% in GG_ND0% to over 37% for GG_ND2%. This increase in porosity is consistent with the improved filament fidelity and reduced shrinkage observed at higher nanofiller concentrations, likely due to the reinforcement effect of NDs increasing the total solid content, stabilizing the hydrogel network against shrinking during gradient ethanol drying, and preventing collapse. In contrast, variations in the average pore area (Figure 4d) were not significant across groups, ranging from 140 µm^2^ for the control group to 230 µm^2^ for the well-defined pores of GG_ND2%. Importantly, this quantification was based on SEM, capturing the top-most layers of material, which are less affected by the deposition of subsequent layers and may present more uniform pore features.

To better understand the internal scaffold architecture, interconnected porosity, and structural deformation under load, µCT was used for the 3D assessment of hydrated scaffolds. This enabled the visualization of the scaffolds before (Figure 4e) and after mechanical compression (Figure 4f), with Figure 4g showing an overlap between undeformed (no color) and compressed (red) structures. Control scaffolds (GG_ND0%) demonstrated numerous irregularities before compression, including closed pore channels. Upon compression, the deformation and lateral expansion were substantial, confirming their low mechanical resilience and insufficient internal reinforcement. Incremental improvements were observed in 0.5% ND loading, with more open porosity and reduced lateral expansion, though deformation remained significant. Notably, scaffolds containing 1% ND displayed better structural coherence, reduced lateral expansion, and improved resistance to mechanical loading. The highest ND loading (GG_ND2%) provided the most effective structural stabilization, demonstrating remarkable preservation of scaffold architecture under compression, with minimal deformation and lateral expansion (Figure 4e). In addition, the porosity remained open before and after compression, with larger and more consistent pores observed in the GG_ND1% and GG_ND2% across scaffold layers. Such pore architectures could be associated with enhanced nutrient permeability, vascular infiltration, bone tissue formation, and ECM production [46].

Rheological characterization was conducted to assess the viscoelastic behavior of the hydrogel formulations across a range of deformations (Figure 4f,g). At a frequency of 1 Hz, when oscillation amplitude was varied (Figure 4f), all samples had a predominantly elastic behavior, with G′ dominating over G″ for most of the tested stress range. This confirms the gel-like behavior of the formulations at low and medium shear stress values. At higher shear, G′ crosses G″, indicating the irreversible structural deformation of the hydrogel network. The yield point (the crossover of G′ and G″) of GG_ND0% and GG_ND0.5% occurs around 400 Pa, shifting to higher values for increased ND concentrations. For GG_ND1%, the irreversible network deformation occurs around 650 Pa, while for GG_ND2%, it is shifted towards 1000 Pa. This suggests enhanced structural strength and network integrity and higher forces needed to induce the irreversible deformation of the hydrogel network at higher ND loadings, likely due to stronger physical interactions between the NDs and the GG polymer chains. When the oscillation frequency was varied, the complex modulus (G*) remained relatively stable across frequencies for all samples, with GG_ND2% showing the least variation. Therefore, the hypothesis that higher ND loading improved the stability of the hydrogel network, minimizing viscoelastic relaxation under oscillatory loading, is further supported. While the rheological differences among groups were modest, the trends could suggest improved hydrogel resilience at higher ND content, which could influence overall scaffold performance post-printing.

Uniaxial compression tests were performed on printed scaffolds in the hydrated state, using similar samples as for µCT imaging. The average compressive modulus measured at 2% strain is illustrated in Figure 4j. Interestingly, in contrast to rheological measurements and µCT-based structural preservation, the compressive stiffness decreased across all ND-containing groups compared to GG_ND0% (approx. 158 kPa). GG_ND0.5% and GG_ND2% showed significantly reduced moduli (65 and 31 kPa, respectively), while GG_ND1% displayed a moderate modulus (105 kPa). The variation in stiffness across ND concentrations reflects a complex interplay between NP reinforcement, nanofiller dispersion, and print fidelity. The compressive modulus is highly influenced by the occurrence of structural defects, closed pore channels, and variations in filament deposition during printing. The reduced stiffness at 0.5% ND loading could also suggest insufficient nanofiller concentration for their homogenous dispersion within the matrix, hindering homogenous load distribution [47]. The non-linear relationship between the compression modulus and the ND loading, particularly in GG_ND1%, could be attributed to the effective NP dispersion within the hydrogel matrix at 1% ND loading, supporting load transfer and promoting local matrix stiffening through possible hydrogen bonding interactions with the polymer chains [48,49]. While GG_ND2% exhibited excellent structural integrity in µCT, the slight decrease in modulus compared to GG_ND1% may indicate early-stage nanoparticle aggregation and structural interference at higher loading, despite the uniform extrusion process and surface uniformity observed in SEM. Such phenomena have been previously reported in NP-reinforced matrices, where both underfilling and overloading can reduce mechanical performance due to aggregation, network interference, or defects introduced during fabrication and processing [47,50,51]. Despite having the lowest modulus among the tested groups, GG_ND2% remains relevant, as soft hydrogel environments in the range of 20–30 kPa have yielded comparable results with the polystyrene substrates in terms of cell migration speed and cell spreading area of MC3T3-E1 preosteoblasts [52]. Additionally, the local nanomechanical properties induced by the presence of NDs may complement biologically relevant cues for preosteoblast attachment and spreading, contributing to cellular responses independently of global scaffold mechanics [53].

### 3.3. Influence of ND Loading on Cellular Behavior

**Figure 5:**
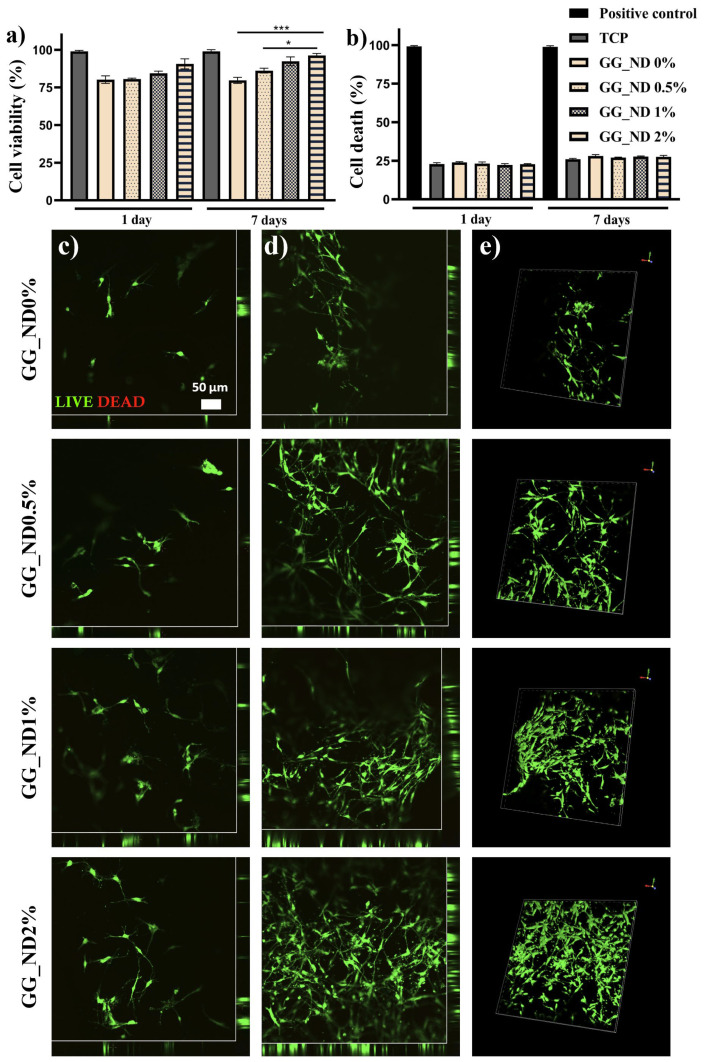
In vitro biocompatibility investigation on the effect of ND loading of GG scaffolds: (**a**) evaluation of MC3T3-E1 metabolic activity using MTT assay; (**b**) cytotoxicity assessment through LDH assay (* *p* < 0.05, *** *p* < 0.005); Live/Dead staining (live cells are represented in green and dead cells are represented in red) of MC3T3-E1 cells in contact with GG_ND scaffolds after (**c**) 1 day and (**d**) 7 days of in vitro culture; (**e**) 3D distribution of cells after 7 days of culture. Scale bar is 50 μm.

To explore whether the structural reinforcement and morphological improvements induced by ND incorporation into the GG matrix also influence biological interactions with the scaffolds, cellular responses were assessed using MC3T3-E1 murine preosteoblasts. Cells were seeded onto casted hydrogel scaffolds (5 mm diameter, 2 mm height) to evaluate viability and proliferation, cytotoxic profile, and cell adhesion over 7 days of culture (Figure 5).

The biocompatibility evaluation of GG_ND scaffolds showed overall satisfactory results, which are represented in Figure 5. Quantitative evaluation of cellular metabolic activity after 1 day of in vitro culture revealed a good viability on all tested composites compared to TCP (98% viability), with a slight increase for the GG_ND2% (90% viability) (Figure 5a). After 7 days of culture, a beneficial effect of ND loading in the GG matrix can be observed. Cells grown on GG_ND0% relatively maintained their metabolic activity (81% viability) compared to TCP from 1 to 7 days of culture. However, for the ND-enriched systems, there was an increase in viability during the period of testing, with the MC3T3-E1_GG_ND2% system having the best results (96% viability), comparable to TCP (98% viability). Regarding the cytotoxic profile, low levels of lactic dehydrogenase release were registered after 1 day of culture for all systems, with a slight increase upon 7 days post-seeding. Additionally, no significant differences were registered between these compositions at both tested time points. LDH assay results (Figure 5b) indicated that the addition of ND to the GG matrix does not induce any significant cytotoxicity to MC3T3-E1 cell culture throughout the 7 days of testing.

The microscopy images acquired after the Live/Dead assay allowed the concomitant observation of live and dead cells in contact with GG_ND substrates after 1 (Figure 5c) and 7 days (Figure 5d) of in vitro culture, as well as the cellular distribution and spreading. Live cells are represented in green and were labelled with calcein AM, and dead cells’ nuclei are represented in red and were labelled with EtBr. After 1 day of culture, green live cells can be seen on all tested composites, with the MC3T3-E1_GG_ND2% system displaying a higher proportion. Additionally, no red nuclei are present on either material, and cells grown on GG_ND1% and GG_ND2% scaffolds present an elongated shape, indicating a good interaction between murine preosteoblasts and ND-enriched composites in the first hours. After 7 days, cell proliferation is evident compared to day 1, especially for the GG_ND2% composite. Moreover, the orthogonal and 3D projections of the systems (Figure 5e) display the cellular distribution after 7 days of culture. GG_ND2% presents not only the highest proportion of live cells but also a more uniform cellular distribution compared to the other composites, which can be attributed to the increased ND content. These observations are highly correlated with the results from the quantitative assays, confirming the positive impact of NDs on murine preosteoblast culture.

**Figure 6:**
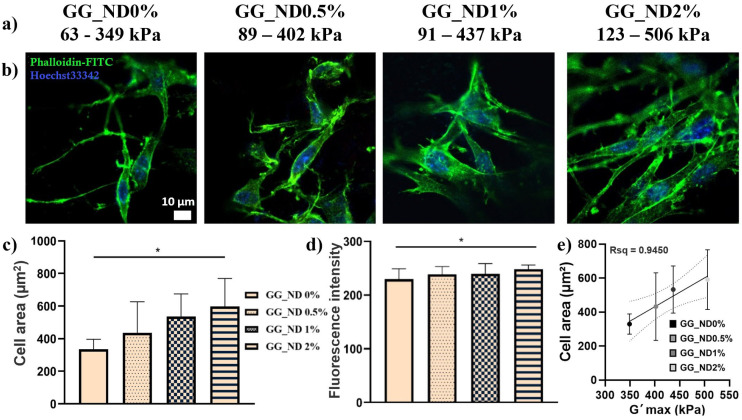
Influence of ND content on local stiffness and MC3T3-E1 preosteoblast adhesion: (**a**) local storage modulus (G′, kPa) measured by nanoindentation on hydrated scaffolds with increasing ND concentrations; (**b**) representative fluorescence microscopy images of MC3T3-E1 cells 48 h post-seeding, stained with phalloidin-FITC (actin filaments, green) and Hoechst 33342 (cells’ nuclei, blue) (* *p* < 0.05); (**c**) quantification of cell spreading area and (**d**) fluorescence intensity of actin filaments; (**e**) correlation between cell area and maximum local storage modulus (G′ max, kPa). Scale bar is 10 μm.

Findings from these biological assays were correlated with the local mechanical properties of the scaffolds’ surfaces, which were investigated through nanoindentation. The panel in Figure 6a shows the minimum and maximum values of the storage modulus (G′, kPa) that were obtained for rehydrated casted scaffolds. A proportional increase in G′ can be observed along with the ND concentration increase, from a maximum of 349 kPa in the absence of NDs to a maximum of over 500 kPa in 2% ND loading, confirming their properties towards GG matrix reinforcement. Correlated with their improved biological response, this is in accordance with other studies stating that early cell adhesion is determined by non-specific cell–biomaterial surface interactions, with surface characteristics such as roughness, chemical functionalization, and surface charge dictating initial cell responses by mediating adhesion and protein adsorption [54].

While compression tests evaluated the overall mechanical behavior of the constructs, nanoindentation assessed localized surface properties at the microscale. The differences observed between these two methods reflect a scale-dependent mechanical response and provide complementary insights. For instance, although the compressive modulus of the GG_ND2% scaffolds remained around 30 kPa, nanoindentation revealed surface stiffness values reaching up to 500 kPa, highlighting the localized reinforcement imparted by NDs. This contrast arises because the bulk mechanical properties are strongly influenced by macrostructural features such as open porosity and architectural fidelity of the scaffolds, whereas nanoindentation captures the mechanical contributions of nanoscale domains, including ND-rich regions localized towards the filament surface. Rather than prioritizing high compressive strength, our approach aimed to fabricate a hydrated, biocompatible matrix, in which NDs act as rigid inclusions that locally reinforce the network and enhance surface stiffness at the cell–material interface.

The wide range of values obtained in nanoindentation illustrates the anisotropic character of the composite structures, as well as the reduced dimensions of the punch (roughly 500 µm), allowing the measurement of particular areas of the filaments. Depending on those two factors, the measurement could reflect the soft hydrated GG matrix, with lower G′ values, or areas of eventual ND agglomerates, with their increased hardness yielding higher storage modulus. The increase in G′ could also be determined by enhanced interactions between the rich surface chemistry of NDs and the GG matrix [55], with possible hydrogen bonding and ionic interactions enabling NDs to act as mild physical crosslinking agents for the GG molecules. Despite their effective initial ultrasonic dispersion, NDs have a natural tendency to agglomerate, due to the functional groups present on their surface. Although it might be viewed as a drawback, this behavior can be of great use. In our previous studies in 2D environments, ND agglomerates localized towards the surface of gelatin substrates enhanced preosteoblast spreading and viability, while in hASCs, ND clusters supported cell adhesion, migration, and proliferation on the surface of electrospun fibers [12]. In addition, F-actin staining showed a proportional increase in hASCs cytoskeleton elongation along with increasing NDs concentrations, supporting their differentiation towards neural-like cells, with most favorable cell responses recorded at 1% *w*/*v* ND loading, compared to 0 or 0.5%.

Here, the influence of ND presence on cellular adhesion was investigated by staining the actin filaments of MC3T3-E1 preosteoblasts with phalloidin-FITC 48 h post-seeding (Figure 6b). Cells adhered to ND-enriched substrates displayed a well-developed cytoskeleton network compared to GG_ND0%, and this effect seems to be enhanced on GG_ND2%, emphasizing the benefits of this nanocomponent in supporting cellular adhesion. Furthermore, cell area measurements (Figure 6c) confirmed that the average cell area increased with ND concentration, with significantly greater spreading observed for GG_ND2% compared to GG_ND0% (*p* < 0.05). A similar trend can be observed in fluorescence intensity (Figure 6d), with higher values registered for the GG_ND2% scaffolds. The increase in fluorescence intensity could be attributed to the development of more defined cytoskeletal networks and possibly thicker actin filaments. Those effects could be linked to the increased local stiffness imparted by ND loading, as it is widely reported that cells grown on stiffer substrates tend to undergo cytoskeleton network reorganization and activation of mechanotransduction pathways that, in the end, promote osteogenic differentiation [56,57,58]. To further support this relationship, cell area values were plotted against the maximum local storage modulus (G′ max) obtained through nanoindentation (Figure 6e). A positive linear correlation was observed (Rsq = 0.9450), suggesting that increased local stiffness at the scaffold surface promotes greater cell spreading. Although global scaffold stiffness measured in uniaxial compression was reduced in GG_ND2%, there is a strong link between local mechanical cues, such as those created by ND-rich regions, and cell behavior at the cell-material interface. Our findings are consistent with other studies that have utilized ND to improve cellular interactions with various types of scaffolds [11,12,13,20]. In poly(L-lactide)-co-(ɛ-caprolactone) copolymer scaffolds, surface functionalization using NDs enhanced cell attachment, proliferation, and differentiation in bone marrow mesenchymal stem cells [20], while in sheep calvarial defects, they determined an increase in BMP-2 and COL I expression, leading to over 20% enhancement of new bone formation compared to non-functionalized scaffolds. The incorporation of ND into poly(ε-caprolactone) fibrous matrices has led to an increase in MC3T3-E1 viability and proliferation and had a stimulatory effect on their osteogenic differentiation by increasing mineralization and the activity of alkaline phosphatase (ALP) [19]. These results underscore the potential of ND inclusion for structural reinforcement of GG matrices, as well as the potential of guiding cell–material interactions through nanomechanical modulation. By creating nanomechanical anchor points at the scaffold surface, ND-rich regions may enhance mechanosensitive cell behaviors such as spreading, adhesion, and cytoskeletal organization.

### 3.4. Bioactivation

Considering the excellent injectability of the GG_ND2% formulation, supporting the fabrication of scaffolds with enhanced morphostructural characteristics that elicit improved cellular responses of MC3T3-E1 preosteoblasts, this formulation was selected for bioactivation using ICA. It was shown that ICA treatment promoted bone mesenchymal stem cells’ viability and osteogenic differentiation by upregulating BMP-2/Smad5 and WNT/B-catenin pathways, increasing osteogenic markers like *Runx2* expression and stimulating ALP activity, both in vitro and in vivo, in a rat femoral head osteonecrosis model [59]. Furthermore, ICA can promote angiogenesis by activating endothelial cell migration and proliferation, which is essential for bone tissue regeneration [60].

In our case, while both GG and NDs are inherently biocompatible, neither the polysaccharide structure of GG nor the inert carbon core of NDs provides specific biochemical cues to promote initial cell adhesion, spreading, and proliferation. To enhance interactions between scaffolds and cells, ICA was incorporated into the GG_ND2% matrix, owing to its well-known osteoinductive and pro-angiogenic properties [61]. Its concentration of 0.1% *w*/*v* was selected based on previous studies demonstrating osteogenic efficacy at this level without inducing cytotoxic effects [22].

**Figure 7:**
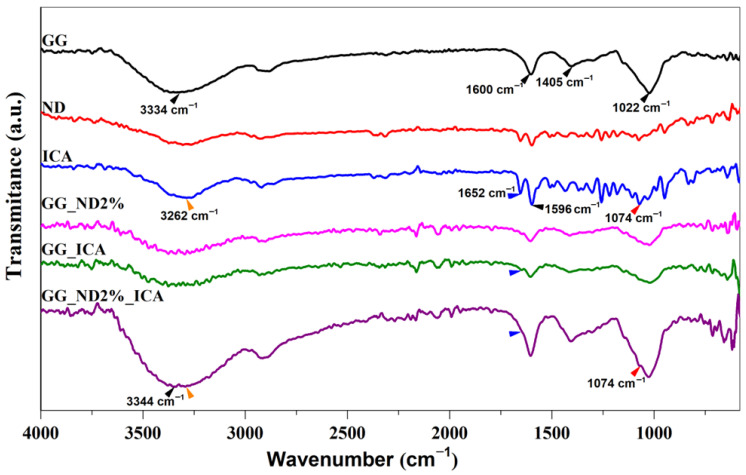
ATR-FTIR spectra showing the compositional characteristics of raw materials (GG, ND, ICA) and of the selected formulations (GG_ND2%, GG_ICA, and GG_ND2%). Orange arrowheads indicate the O-H stretching vibrations in ICA (3262 cm^−1^) and in GG_ND2%_ICA (shifted to 3344 cm^−1^); blue arrowheads mark the ketonic C=O stretching vibrations of ICA and its preserved presence in GG_ICA and GG_ND2%_ICA; red arrowheads correspond to the C-O-C vibration observed both in ICA and GG_ND2%_ICA.

The structural characteristics and potential interactions of the selected formulations (GG_ND2%, GG_ICA, and GG_ND2%_ICA) were evaluated by ATR-FTIR using the precursor materials as control (Figure 7). In the spectrum of pure GG, the broad band centered at 3334 cm^−1^ corresponds to O-H stretching vibrations, characteristic of polysaccharide hydroxyl groups. Additional peaks at 1600 cm^−1^ and 1405 cm^−1^ can be attributed to the asymmetric and symmetric stretching vibrations of carboxylate groups, respectively, while the band at 1022 cm^−1^ is related to C-O deformation vibrations [62]. The spectra of ND and ICA powders showed characteristic bands in the 3400–3100 cm^−1^ region, with peaks around 3260 cm^−1^ assigned to their O-H stretching vibrations [26]. ICA showed a more prominent peak at 1652 cm^−1^, corresponding to the ketonic C=O stretching vibration, and two other specific peaks at 1569 cm^−1^ (likely due to aromatic C=C stretching vibration) and 1074 cm^−1^ (corresponding to the C-O-C stretching vibration) [18,26]. Upon incorporation of NDs and ICA within the GG matrix, a decrease in the intensity of the O-H stretching band was observed, suggesting potential hydrogel bonding and other physical interactions between the formulation components. In the C=O vibration region, this characteristic band is retained in both GG_ICA and GG_ND2%_ICA (blue arrowheads, Figure 7), suggesting the preservation of ICA’s carbonyl functionality within the matrix. The GG_ND2%_ICA formulation showed the most pronounced O-H stretching band, slightly shifted to 3344 cm^−1^, indicating enhanced hydrogen bonding interactions between the surface of NDs previously decorated with ICA, followed by interactions with glucopyranose units of GG powder and potentially unmodified NDs. This O-H band broadening and increased intensity, along with its slight shift compared to the spectra of ICA (marked in orange arrowheads) and the retention of the peak at 1074 cm^−1^ (red arrowheads), are indicative of increased hydrogen bonding between ICA and ND particles and confirm the presence of ICA in the composite formulation.

**Figure 8:**
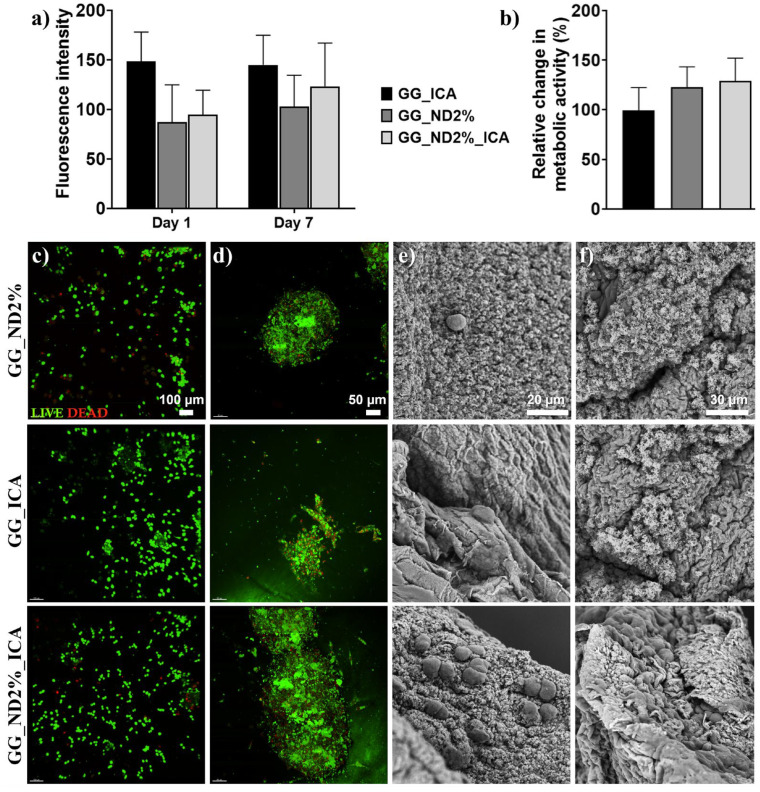
Effect of ICA functionalization on MG-63 viability and surface mineralization of GG_ND2% scaffolds: (**a**,**b**) quantification of MG-63 cell viability and metabolic activity on GG_ND2%, GG_ICA, and GG_ND2%_ICA scaffolds, showing (**a**) fluorescence intensity from Live/Dead staining on days 1 and 7; (**b**) relative change in metabolic activity at day 7 compared to day 1; (**c**,**d**) Live/Dead staining of MG-63 cells cultured on scaffolds after (**c**) 1 day and (**d**) 7 days of in vitro culture; (**e**,**f**) representative SEM images of scaffold surfaces after (**e**) 1 day and (**f**) 7 days of culture. Scale bars: 100 µm (**c**), 50 µm (**d**), 20 µm (**e**), and 30 µm (**f**).

Regarding biological interactions, the comparative analysis of GG_ND2% (negative control), GG_ICA (positive control), and GG_ND2%_ICA scaffolds (casted hydrogel discs, 5 mm diameter, 2 mm height) revealed different effects of ND reinforcement and ICA bioactivation on MG-63 cell behavior. The quantitative analysis of cell viability using fluorescence intensity (Figure 8a) showed that all groups maintained relatively stable fluorescence values between day 1 and day 7. While the GG_ICA group showed slightly higher fluorescence on day 1, no statistically significant differences were observed among the groups over time. This suggests that neither ND reinforcement nor ICA incorporation had cytotoxic effects on MG-63 cells during the 7-day period, further confirming their cytocompatibility. The 1% (*v*/*v*) DMSO concentration used in the ICA-containing formulations was selected to ensure ICA solubilization without compromising cell health [63].

Metabolic activity, evaluated using relative changes between day 1 and day 7 (Figure 8b), showed an upward trend across all groups, with slightly higher values observed for GG_ND2%_ICA. Although these differences were not statistically significant, the enhanced metabolic activity in ICA-containing scaffolds may reflect early cellular activation and improved functional status, which could precede more robust osteogenic outcomes. Live/Dead staining images (Figure 8c,d) show that while all groups maintained high cell viability after 1 day (Figure 8c) and 7 days (Figure 8d) of culture, the presence of ICA not only did not compromise cytocompatibility, but it also enhanced cytocompatibility and cell distribution on the scaffold surface. Previous reports suggest that flavonoids like ICA can physically adsorb on carbonaceous nanoparticles through hydrogen bonding, with numerous hydroxyl groups in ICA and ND chemical functionalities being able to enhance cell response to the hydrogels’ surface [22]. We observed that GG_ND2% scaffolds showed apparent lower cell viability and more pronounced cell clustering, compared to ICA-containing formulations. In contrast, ICA-containing scaffolds (GG_ICA and GG_ND2%_ICA) appeared to support better cell dispersion, with fewer dead cells compared to the negative control. GG_ND2%_ICA scaffolds displayed the most uniform cell distribution, with moderate elongation after 7 days, which was indicative of enhanced cell–matrix interactions.

In previous findings, combinations of ICA with different nanocarriers have been shown to stimulate osteoblast function and promote bone regeneration. Choi et al. [18] demonstrated that ICA-functionalized NDs significantly enhanced osteogenic differentiation of preosteoblasts, increasing ALP activity, mineral deposition, and expression of osteogenic markers, confirming the potential of ND-ICA composites for bone regeneration applications. Similarly, Lai et al. [21] reported that a porous ICA-loaded poly(lactic-co-glycolic acid)/β-calcium phosphate scaffold promoted bone regeneration in an osteonecrotic rabbit model, with ICA facilitating MC3T3-E1 cells’ growth into the scaffolds. In addition, they observed accelerated matrix mineralization and neovascularization, emphasizing ICA’s osteogenic and angiogenic effects.

SEM micrographs confirmed enhanced cell–matrix interactions, showing surface mineralization after 1 day of culture (Figure 8e), which was more predominantly on scaffolds containing NDs, along with extracellular matrix (ECM) deposition visible after 7 days (Figure 8f). On GG_ND2%, cells appeared more rounded, with limited filopodial extensions after 7 days. GG_ICA scaffolds promoted slightly higher cell flattening and early ECM deposition. On the GG_ND2%_ICA scaffold, cells exhibited more extensive spreading after 7 days, with broad lamellipodia spreading on the hydrogel surface and numerous spherical mineral-like nodules coating the scaffold surface, suggesting accelerated matrix mineralization.

These findings point to a complementary effect—while NDs structurally reinforced the scaffold and promoted stiffness-driven cytoskeletal organization, ICA enhanced cell-scaffold interactions. Its pro-angiogenic effects may also contribute to enhanced cell migration and survival within the pore channels of our 3D-printed structures. While these observations are promising, further investigation is required to confirm the individual and combined contributions of these components under physiologically relevant conditions. Several limitations of this study should be considered in this context.

Given ICA’s role in osteogenesis and angiogenesis, its release kinetics are an important determinant of the scaffolds’ long-term bioactivity. Although a detailed ICA release study was not conducted in this work, several reports have demonstrated that ICA can be successfully loaded into 3D-printed scaffolds via physical adsorption or dispersion, leading to sustained release profiles and bioactive performance. For instance, ICA-loaded PCL and tricalcium phosphate (β-TCP), as well as PVA- β-TCP scaffolds, exhibited extended release over several weeks, with sustained osteogenic effects both in vitro and in vivo [64,65]. Likewise, 3D-printed structures based on silk fibroin and mesoporous bioactive glass NPs (MBGNs) with adsorbed ICA showed controlled release over 24 days and promoted osteogenic differentiation in bone mesenchymal stem cells [66]. Enhanced early osteogenic markers were observed during the first 7 days of cell culture, with pronounced effects in silk fibroin-ICA composites. When adsorbed onto the MBGNs, ICA had a steadier release, with late osteogenic markers significantly more pronounced after 14 days, compared to the silk fibroin-ICA composites, demonstrating that its physical adsorption on the NPs can provide a controlled release and sustain osteogenic differentiation over longer periods of time.

Similarly, in our case, ICA was physically incorporated onto ND surfaces through hydrogen bonding and other physical interactions and embedded in the GG hydrogel matrix. These interactions, together with the dense GG network, are expected to prolong ICA release and minimize burst release, supporting sustained delivery. While this hypothesis requires validation through dedicated release studies, the promising precedents and the structural integrity observed in our constructs suggest favorable release profiles. Since no similar studies have been conducted on ICA-functionalized NDs embedded in 3D matrices, future work will include detailed quantification of ICA release kinetics and scaffold degradation to fully understand their long-term bioactivity.

Moreover, as GG is ionically crosslinked with calcium ions, its gradual degradation under physiological conditions may be accompanied by calcium ion leaching, which could further influence the local microenvironment by introducing pro-regenerative cues. This phenomenon is in ionically crosslinked polysaccharide networks, where Ca^2+^ ions can gradually dissociate from the network due to ion exchange and hydrolytic degradation, leading to particular ion release profiles [67,68]. Further, Ca^2+^ ion leaching could support bone regeneration by promoting osteogenic differentiation, angiogenesis, and mineralization [69]. Given the sensitivity of osteogenic responses to both biochemical and structural cues, evaluating cell–material interactions within architecturally relevant environments is an important next step. In addition, further investigations into the osteoinductive potential of the composite scaffolds should include the quantification of key osteogenic markers such as ALP, *Runx2*, osteocalcin, and osteopontin, to confirm and expand upon the plausible mechanistic link between ND-induced stiffening, ICA bioactivity, and the cellular responses observed in this study.

Together, the integration of NDs and ICA into the GG matrix resulted in multifunctional scaffolds with favorable physicochemical properties and mechanical characteristics. By addressing both the mechanical and biochemical requirements for bone tissue regeneration, this strategy demonstrates the potential of ND/ICA-functionalized inks to bridge the gap between structural biomaterials and bioactive scaffolds.

## 4. Conclusions

In this study, we successfully developed injectable, nanostructured inks based on a bioinert GG matrix loaded with low concentrations of ND particles and bioactivated with ICA. To our knowledge, this is the first study to co-deliver ND-based structural reinforcements and ICA bioactive molecules within 3D-printed GG scaffolds. While previous work has demonstrated the osteogenic potential of ICA-loaded NDs in simple nanoparticle systems or 2D substrates [18,26], our approach integrates these components into a shear-thinning, bioinert, printable hydrogel matrix. This dual functionality enhanced both printability and biological performance, improving cell spreading and mineral deposition. Rheological investigations demonstrated that ND incorporation improved the filament formation properties of GG-based formulations, enabling smooth and reliable extrusion at higher concentrations (1 and 2%), as supported by both rheological measurements and CFD modeling based on power law fitting.

Microstructural analyses illustrated the structural reinforcing effect of ND particles on the GG network, significantly improving filament definition, pore uniformity, and mechanical stability upon drying and rehydration. Compression tests confirmed that increasing ND concentrations notably reduced scaffold deformation and lateral expansion under mechanical stress, with maintained filament fidelity and pore openness, which are necessary for vascular infiltration and nutrient diffusion. Scaffolds containing 2% NDs maintained the highest structural integrity, demonstrating minimal deformation and preserving open pore architectures, which are essential for nutrient transport and tissue ingrowth. Interestingly, while global stiffness decreased at 2% ND content, local nanomechanical reinforcement at the cell interface appeared to drive enhanced cellular responses. Biological assessments revealed that increasing ND concentration significantly enhanced preosteoblast viability, proliferation, and attachment in MC3T3-E1 cells, correlating with improved local stiffness confirmed by nanoindentation. Scaffolds with 2% ND loading showed improved cellular interactions and distribution, as well as cytoskeletal organization, compared to lower nanofiller loadings (0–1%). Bioactivation of this formulation with ICA further improved these interactions, resulting in increased cellular proliferation and more uniform cell distribution across the scaffold surface. Together, the local mechanical reinforcement induced by ND loading and the biochemical stimulation imparted by ICA lead to enhanced cell-scaffold interactions and ECM mineral deposition for the bioactivated formulation.

Compared to earlier 2D studies, in which NDs enhanced cellular spreading and viability on gelatin nanofibers [11,12,13], this study demonstrated that the beneficial cellular interactions are preserved or enhanced within hydrated 3D scaffold environments. The consistent results across both 2D and 3D platforms and various cell types underline the intrinsic ability of NDs to mechanically and biochemically influence cellular behavior in this case, particularly when bioactivated with ICA. However, this study is limited by its short-term in vitro evaluation, lacking long-term degradation analysis and in vivo validation. Future studies will focus on comprehensive in vitro and in vivo testing, including assessment of scaffold biodegradation, ICA release kinetics, and long-term cellular responses. In particular, a critically sized calvarial defect model in rodents could be a relevant established in vivo platform to evaluate osteoconductivity, vascularization, and scaffold remodeling in a physiologically representative environment. Additionally, exploring alternative ND surface functionalization strategies may further enhance bioactivity and allow for more precise control over the regenerative process. Overall, this study lays the groundwork for 3D multifunctional GG—ND—ICA composite scaffolds as promising candidates for bone tissue engineering applications, providing localized mechanical cues and a bioactive microenvironment that could support bone tissue regeneration.

## Data Availability

The original contributions presented in this study are included in the article. Further inquiries can be directed to the corresponding author.

## Abbreviations

The following abbreviations are used in this manuscript:

ND(s)	Nanodiamond(s)
GG	Gellan gum
ICA	Icariin
ECM	Extracellular matrix
hASCs	Human adipose stem cells
3ITT	Three-interval thixotropy test
CFD	Computational fluid dynamics
DW	Distilled water
LVR	Linear viscoelastic region
SEM	Scanning electron microscopy
µCT	High-resolution computerized micro-tomography
DCM II	Dynamic Contact Module II
DMEM	Dulbecco’s Modified Eagle Medium
FBS	Fetal bovine serum
TCP	Tissue culture plastic
MTT	Methylthiazolyldiphenyl tetrazolium bromide
PBS	Phosphate saline buffer
LDH	Lactic dehydrogenase
EtBr	Ethidium bromide
PFA	Paraformaldehyde
DMSO	Dimethylsulfoxide
ATR-FTIR	Attenuated total reflectance Fourier-transform infrared spectroscopy
CM	Complete medium
P/S	Penicillin/streptomycin
DPBS	Dulbecco’s Phosphate-Buffered Saline
ALP	Alkaline phosphatase
